# Evolving evidence on a link between the ZMYM3 exceptionally long GA-STR and human cognition

**DOI:** 10.1038/s41598-020-76461-z

**Published:** 2020-11-10

**Authors:** H. Afshar, S. Khamse, F. Alizadeh, A. Delbari, R. Najafipour, A. Bozorgmehr, M. Khazaei, F. Adelirad, A. Alizadeh, A. Kowsari, M. Ohadi

**Affiliations:** 1 Iranian Research Center on Aging, University of Social Welfare and Rehabilitation Sciences, Tehran, Iran; 2 Department of Genomic Psychiatry and Behavioral Genomics (DGPBG), Roozbeh Hospital, School of Medicine, Tehran University of Medical Sciences (TUMS), Tehran, Iran; 3 Cellular and Molecular Research Centre, Research Institute for Prevention of Non Communicable Disease, Qazvin University of Medical Sciences, Qazvin, Iran; 4 Iran Psychiatric Hospital, Iran University of Medical Sciences, Tehran, Iran; 5 Department of Health Education and Promotion, Faculty of Health Sciences, Tabriz University of Medical Sciences, Tabriz, Iran; 6 Medical Microbiology Research Center and Microbiology Department, Qazvin University of Medical Sciences, Qazvin, Iran; 7 Health Management and Social Development Research Center, Golestan University of Medical Sciences, Gorgan, Iran

**Keywords:** Evolution, Genetics, Molecular biology, Neuroscience

## Abstract

The human X-linked zinc finger MYM-type protein 3 (*ZMYM3*) contains the longest GA-STR identified across protein-coding gene 5′ UTR sequences, at 32-repeats. This exceptionally long GA-STR is located at a complex string of GA-STRs with a human-specific formula across the complex as follows: (GA)8-(GA)4-(GA)6-(GA)32 (ZMYM3-207 ENST00000373998.5). *ZMYM3* was previously reported among the top three genes involved in the progression of late-onset Alzheimer’s disease. Here we sequenced the *ZMYM3* GA-STR complex in 750 human male subjects, consisting of late-onset neurocognitive disorder (NCD) as a clinical entity (n = 268) and matched controls (n = 482). We detected strict monomorphism of the GA-STR complex, except of the exceptionally long STR, which was architecturally skewed in respect of allele distribution between the NCD cases and controls [F (1, 50) = 12.283; p = 0.001]. Moreover, extreme alleles of this STR at 17, 20, 42, and 43 repeats were detected in seven NCD patients and not in the control group (Mid-P exact = 0.0003). A number of these alleles overlapped with alleles previously found in schizophrenia and bipolar disorder patients. In conclusion, we propose selective advantage for the exceptional length of the *ZMYM3* GA-STR in human, and its link to a spectrum of diseases in which major cognition impairment is a predominant phenotype.

## Introduction

Human-specific characteristics and phenotypes such as late-onset neurocognitive disorder (NCD) (also known as dementia) are likely to be the consequence or by-product of human-specific evolutionary events. In agreement with the above model, recent emerging evidence indicates that signals of brain evolution in anatomically modern humans are strongly related to the Alzheimer disease (AD) pathways^1^. Remarkably, certain human-specific derived alleles protect against post-reproductive cognitive decline^2^.

In comparison to single nucleotide substitutions, short tandem repeats (STRs) offer a significantly more versatile reservoir of genetic variations that may be necessary for speciation and species-specific phenotypes^3^. Following a genome-scale analysis of all human protein- coding genes annotated in the GeneCards database, we previously reported a catalog of genes containing “exceptionally long” STRs (> 5 repeats) in their core promoters^4,5^ and the 5′ untranslated region (UTR)^6^. The emerging comparative and functional analyses of a number of the identified STRs support adaptive evolutionary patterns for the expansion of a number of these STRs^3,7^, and the co-occurrence of alleles at the extreme ends of these STRs with major human cognitive disorders, including schizophrenia (SCZ), bipolar disorder (BPD) and late-onset NCD^8–12^ .In line with the above findings, recent reports indicate that STR length influences expression quantitative trait loci (eQTL) associations^13^.

In the category of GA-STRs, the zinc finger MYM-type containing 3 (*ZMYM3)* gene contains the longest annotated 5′ UTR GA-STR at 32-repeats^6^, which is part of a complex of four consecutive GA-STRs of human-specific formula across the complex (ZMYM3-207 ENST00000373998.5) (Table 1)^8^. *ZMYM3* is located at Xq13.1, and encodes a zinc-finger protein, which is a component of histone deacetylase-containing multiprotein complexes that function through modifying chromatin structure to keep genes silent^14^.

**Table 1:** Across-species landscape of the *ZMYM3* GA-STR complex.

Species	GA-STR complex formula
Human	8-4-6-32
Bonobo	5-4-6-11
Chimpanzee	5-4-6-12-16
Orangutan	4-4-4-13
Drill	5
Olive baboon	5-4-4
Macaque	5-4
Golden snub-nosed monkey	5-4-5
Marmoset	4-4-4-18
Bushbaby	–
Capuchin	4
Black snub-nosed monkey	–
Tarsier	4
Mouse Lemur	4-4
Chinese hamster	–
Ferret	8-4-15
Guinea pig	–
Platypus	–
Panda	11-4-4
Goat	–
Lion	8-5-5
Elephant	–
Mouse	–
Arabian camel	–
Armadillo	–
Cat	6-4-6
Dog	20
Mega bat	10-5
Rabbit	6
Cow	–
Chicken	–

*ZMYM3* was previously reported among the top three genes involved in the progression of late-onset AD^15^. Several alternatively spliced transcript variants have been found for this gene, of which the variant containing the exon 1 5′UTR is specifically expressed in the brain^16^, and spans the GA-STR complex^6^. Disruption of this GA-STR complex was reported in a X:13 translocation in a case of X-linked mental retardation^17^. More recently, deleterious mutations in the coding sequence of this gene were reported in conjunction with X-linked intellectual disability in a Finish family by X-exome sequencing^18^.

Here we sequenced the *ZMYM3* GA-STR complex in late-onset neurocognitive disorder (NCD) patients and matched controls. This investigation was founded on the following facts: the role of *ZMYM3* as one of the top three genes involved in the progression of late-onset AD, exceptional length of the STR in human and human-specificity of the STR complex formula in which this STR is located, a link between this STR and instances of cognition deficit (a property that is severely compromised in NCD), its predominant expression in the human brain, and proximity to the + 1 TSS.

## Materials and methods

### Subjects

Seven hundred fifty unrelated Iranian male subjects (age ≥ 60 years), consisting of late-onset NCD patients (n = 268) and controls (n = 482) were recruited from the provinces of Qazvin and Rasht. All patients were included based on the Diagnostic and Statistical Manual of Mental Disorders (DSM-5) for NCD. In each participant, the Persian version of the Abbreviated Mental Test Score (AMTS) was implemented (inclusion criteria: AMTS < 7), medical history was taken, complemented by CT-scans in a number of instances (Suppl. 1). The control group was selected based on AMTS of ≥ 8, and history in all subjects, and normal CT-scan where possible. The AMTS is currently one of the most accurate primary screening instruments to increase the probability of NCD^20^. The Persian version of the AMTS is a valid cognitive assessment tool for older Iranian adults and can be reliably used for NCD screening in Iran, with an over 90% sensitivity^19^. The cases and controls were matched based on age and residential district. Informed consent was obtained from the subjects (informed consent was obtained from the guardians of all the subjects where necessary) and their identities remained confidential throughout the study. This research was approved by the Ethics Committee of the University of Social Welfare and Rehabilitation Sciences, Tehran, Iran, and was consistent with the principles outlined in an internationally recognized standard for the ethical con duct of human research.

### Statistical analysis

The chi-squared test was used to compare the distribution of each allele between the control and NCD groups. The Mid-P exact test was used for the alleles detected at the extreme ends of the allele distribution curve, which were detected in the NCD patients, and not in the controls in this study and two previous studies of SCZ and BPD^8,9^. Levene's test was used to assess the equality of variances of allelic distribution for the two groups.

### Allele/genotype analysis of the *ZMYM3* gene GA-STR complex

Genomic DNA was obtained from peripheral blood using a standard precipitation method, and PCR was carried out as previously described^8^. Briefly, PCRs were performed in a thermocycler (peqSTAR) under the following conditions: 94 °C for 4 min, followed by 40 cycles including denaturing at 94 °C for 30 s, annealing for 30 s at 63 °C, and extension at 72 °C for 30 s. A final extension was conducted at 72 °C for 5 min. All samples were sequenced for the *ZMYM3* GA-complex using an ABI PRISM 377 DNA sequencer.

## Results

### Variation status of the *ZMYM3* GA-STR complex in the human subjects studied

The complex in which the exceptionally long GA-STR (32-repeat) is located consists of four consecutive GA-STRs with the 8-4-6-32 formula in human^8^. The 8-4-6 formula was found to be monomorphic across the 750 human subjects studied. The exceptionally long GA-STR, however, was polymorphic in the human subjects studied. The 8-4-6-32 formula was human-specific when screened in 31 species encompassing various orders, including Primates, Rodents, Laurasiatheria, Scandentia, and Afrotheria (Table 1).

### Alteration of the overall allele/genotype architecture at the exceptionally long GA-STR in late-onset NCD patients vs. controls

**Figure 1:**
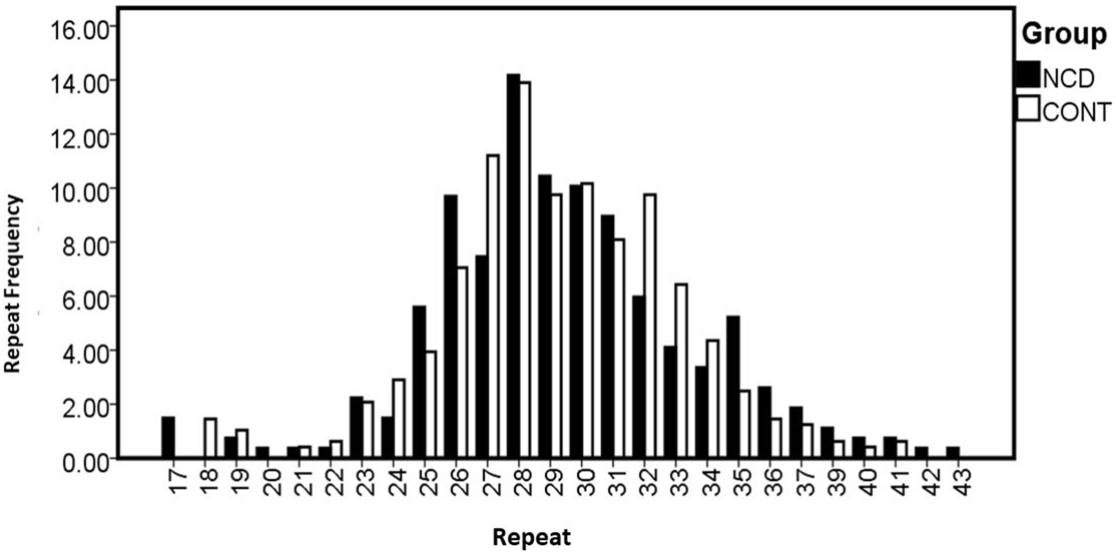
Allele range of the *ZMYM3* GA-repeat in the human subjects studied (cases and controls included).

The overall distribution of the alleles was compared between the NCD cases and controls, which revealed inequality of the population variances between the two groups [F (1, 50) = 12.283; p = 0.001] (Fig. 1).

### Allele range of the *ZMYM3* GA-repeat in the late-onset NCD patients and controls

The allele range of the GA-repeat was between 18 and 41-repeats across the control subjects, and 17 and 43-repeats in the late-onset NCD patients (Fig. 1, Table 2).

**Table 2:** Allele/genotype distribution of the *ZMYM3* exceptionally long STR in NCD patients and controls.

Repeat size	NCD	CONT	Chi square	P-value
17	4	0	7.233**	0.00715755
18	0	7	3.929*	0.04746016
19	2	5	0.158	0.69100458
20	1	0	1.801	0.17959165
21	1	2	0.008	0.92873007
22	1	3	0.202	0.65311132
23	6	10	0.022	0.8820871
24	4	14	1.466	0.22597787
25	15	19	0.374	0.54083196
26	26	34	0.901	0.34251372
27	20	54	2.71	0.09972099
28	38	67	0.011	0.91647033
29	28	47	0.093	0.76039737
30	27	49	0.002	0.96432941
31	24	39	0.167	0.68279188
32	16	47	3.2	0.07363827
33	11	31	1.764	0.18412637
34	9	21	0.447	0.50376306
35	14	12	3.848*	0.04980535
36	7	7	1.264	0.2608953
37	5	6	0.459	0.4980917
39	3	3	0.536	0.4640952
40	2	2	0.356	0.55073617
41	2	3	0.04	0.84148058
42	1	0	1.801	0.17959165
43	1	0	1.801	0.17959165
Total	268	482		

*NCD *neurocognitive disorder, *CONT *control.

**p < 0.01, *p < 0.05.

### Disease-only alleles across the *ZMYM3* exceptionally long GA-STR in late-onset NCD patients

**Figure 2:**
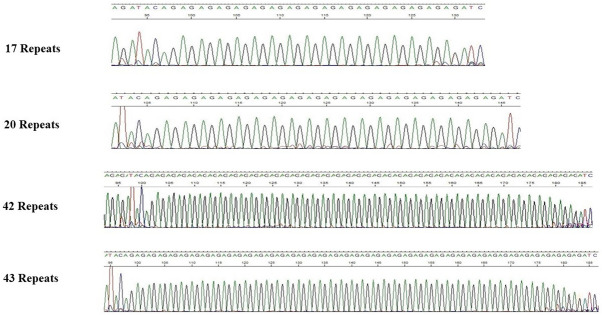
Electropherogarm of the ZMYM3 GA-repeat extreme alleles in the NCD patients.

Alleles were detected at 17, 20, 42, and 43-repeats in seven NCD patients (2.61% of the NCD cases) that were not detected in the control individuals (Mid-P exact = 0.0003) (Fig. 2). On the other hand, all alleles that were detected in the controls were also detected in the NCD patients. The 17-repeat is the shortest allele detected in our human samples to date. The longest allele detected in human to date is at 45-repeats, detected in a case of SCZ^8^.

### Clinical characteristics of the patients harboring disease-only alleles

**Figure 3:**
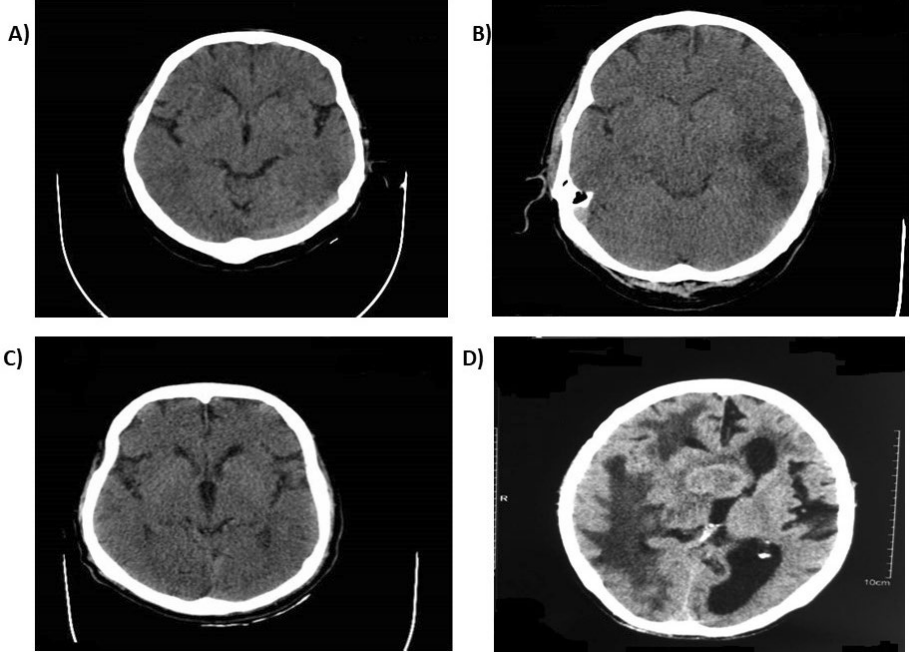
CT-scan of the patients with the extreme alleles. (**A**) 17-repeat, (**B**) 17-repeat, (**C**) 42-repeat, (**D**) 43-repeat. Extensive hypodense areas and calcification were detected in various brain sections.

**Figure 4:**
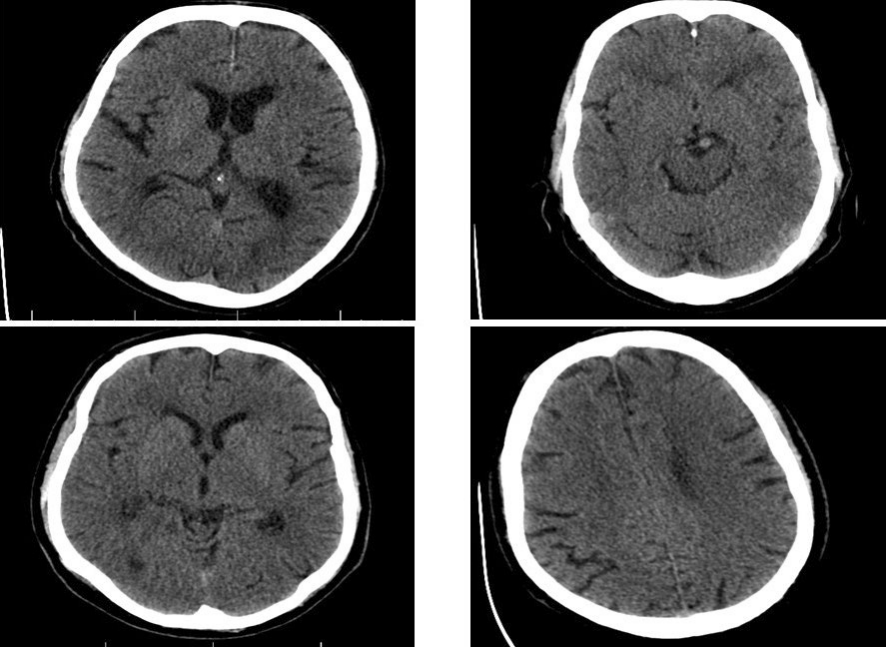
CT-scan of a number of control individuals.

The seven patients harboring disease-only alleles (Table 3) revealed extensive abnormalities in the available CT-scan records (Fig. 3). The observed lesions included extensive hypodense areas, calcifications, cortical atrophy, and ventricular enlargement. In patients, 2, 3, and 4, bilateral periventricular hypodense areas were detected, which indicated possible chronic microvascular changes and vascular dementia. The remaining four patients may be having AD based on the gradual deterioration of cognition in clinical examination and extensive temporal and cortical atrophy. CT-scans of a number of control individuals are also included for comparison (Fig. 4).

**Table 3:** NCD patients harboring alleles at the extreme ends of the *ZMYM3* exceptionally long GA-STR*.

Patient no.	Age	STR repeat	AMTS**
1	93	17	5
2	64	17	4
3	65	17	1
4	65	17	5
5	83	20	5
6	80	42	6
7	78	43	5

*Those alleles were not detected in our NCD cohort and two cohorts previously studied, including schizophrenia and bipolar disorder.

**Abbreviated mental test score.

## Discussion

The *ZMYM3* GA-STR complex is human-specific in formula, the exceptionally long STR within this complex reaches maximum length in human, and the transcript encompassing this STR complex is specifically expressed in the brain (Table 1). ZMYM3 was previously reported among the top three regulators of AD progression^15^. This gene may also link to other major disorders that are associated with major cognition impairment in human, such as SCZ, BPD, and intellectual disability^8,9,17,18^. The above findings raise the possibility that *ZMYM3* may be a master gene in the evolution of human cognition.

We investigated the *ZMYM3* GA-STR complex in late-onset NCD as a clinical entity, without differentiating the subtypes of NCD. The advantage of this novel approach was to eliminate the often-ambiguous diagnoses made for the NCD subtypes, which frequently co-occur and overlap in respect of the clinical and pathophysiological manifestations^9,21–25^.

We found a significant skewing of the genetic architecture at the exceptionally long STR in the NCD patients vs. controls. Moreover, in seven NCD patients, we detected alleles that were not detected in the controls. A number of the disease-only alleles overlapped with alleles detected previously by our group in SCZ and BPD^9^. The 17 and 43-repeat alleles overlapped in NCD, SCZ and BPD, whereas, the 20-repeat allele overlapped in NCD and SCZ. It is possible that these alleles coincide with the overlapping cognition impairment component across the studied disorders. In line with the above findings, several other genes of overlapping nature have been reported by other groups across late-onset NCD and psychiatric disorders^26–28^. The 42-repeat allele detected in the NCD patients was not detected in any human subjects studied to date.

Although the disease-only alleles detected by our group are at frequencies of < 0.02 in late-onset NCD, and encompass 2.61% of the patients, it is conceivable that fractional numbers of the remaining majority harbor alleles at unknown STR loci yet to be identified in the future studies. It is often claimed that genes affecting health in older age are beyond the reach of natural selection. However, findings on the *APOE* alleles and several other NCD susceptibility loci indicate that natural selection indeed happens in such alleles^29,30^.

The reason we chose only male subjects is that *ZMYM3* is X-linked, and therefore, it is expected that there are significant phenotypic differences as a result of gender. A future study is warranted to explore the significance of this STR in female subjects. Expansion of certain STR classes, especially trinucleotide repeats, can cause a range of neurological disorders, including Huntington disease, various ataxias, motor neuron disease, frontotemporal dementia, and fragile X syndrome^31^. It remains to be clarified how the *ZMYM3* GA-STR complex functions in the human brain. From what we know so far, GA-STRs of the range observed in the *ZMYM3* complex can dramatically alter gene expression^32,33^. ZMYM3 is among the top three master regulators causally responsible for regulating the transcriptional signature of AD progression^15^. While GWAS approaches employ single nucleotide polymorphisms rather than STRs, and therefore can fail to detect instances of association with STRs, they have linked *ZMYM3* to a number of neurological disorders of major cognitive impairment, including prion disease and multiple sclerosis^34,35^.

Considering that the *ZMYM3* GA-complex contains three of the longest GA-STRs identified in a human protein-coding gene 5′ UTR, this GA-rich region may also function as a X chromosome dosage compensation mechanism as described in model organisms such as *Drosophila*^36^. In human, the GAGA-binding c-Krox/Th/POK protein specifically binds to (GA)8^37^, and can modulate chromatin remodeling and gene expression activity. Of note, (GA)8 is one of the human-specific length STRs across the *ZMYM3* STR complex.

## Conclusion

In conclusion, the *ZMYM3* GA-STR is a prime example in which alleles at the extreme short and long ends of exceptionally long STRs may be associated with a spectrum of major human disorders in which cognition impairment is the predominant phenotype. Independent studies of various neurocognitive disorders are warranted to confirm the significance of our findings.

## Supplementary information

Supplementary Figure.

Supplementary Caption.

## Abbreviations

AD: Alzheimer’s disease
BPD: Bipolar disorder
NCD: Neurocognitive disorder
SCZ: Schizophrenia
STR: Short tandem repeat
TSS: Transcription start site
UTR: Untranslated region
ZMYM3: Zinc finger MYM-type protein 3
