# Supplementary material for: Evolving evidence on a link between the ZMYM3 exceptionally long GA-STR and human cognition
Source: Sci Rep. 2020 Nov 10;10:19454. doi: 10.1038/s41598-020-76461-z (PMC7655811; doi:10.1038/s41598-020-76461-z)
Supplement: Supplementary file 1 — Supplementary Figure. [file 41598_2020_76461_MOESM1_ESM.pdf]

Evolving evidence on a link between the ZMYM3 exceptionally long GA-STR and human cognition.

Afshar H[1], Khamse S[1], Alizadeh F[2], Delbari A[1], Najafipour R[3], Bozorgmehr A[4], Khazaei M[1], Adelirad F[5], Alizadeh A[6], Kowsari A[7], Ohadi M[1]*

1- Iranian Research Center on Aging, University of Social Welfare and Rehabilitation Sciences, Tehran, Iran.

2- Department of Genomic Psychiatry and Behavioral Genomics (DGPBG), Roozbeh Hospital, School of Medicine, Tehran University of Medical Sciences (TUMS), Tehran,
Iran.

3- Cellular and Molecular Research Centre, Research Institute for Prevention of Non Communicable Disease, Qazvin University of Medical Sciences, Qazvin, Iran.

4- Iran Psychiatric Hospital, Iran University of Medical Sciences, Tehran, Iran.

5- Department of Health Education and Promotion, Faculty of Health Sciences Tabriz University of Medical Sciences, Tabriz, Iran

6. Medical Microbiology Research Center and Microbiology Department, Qazvin University of Medical Sciences, Qazvin, Iran.

7. Health Management and Social Development Research Center, Golestan University of Medical Sciences, Gorgan, Iran.

*Corresponding author

E-mail Address: ohadi.mina@yahoo.com
mi.ohadi@uswr.ac.ir

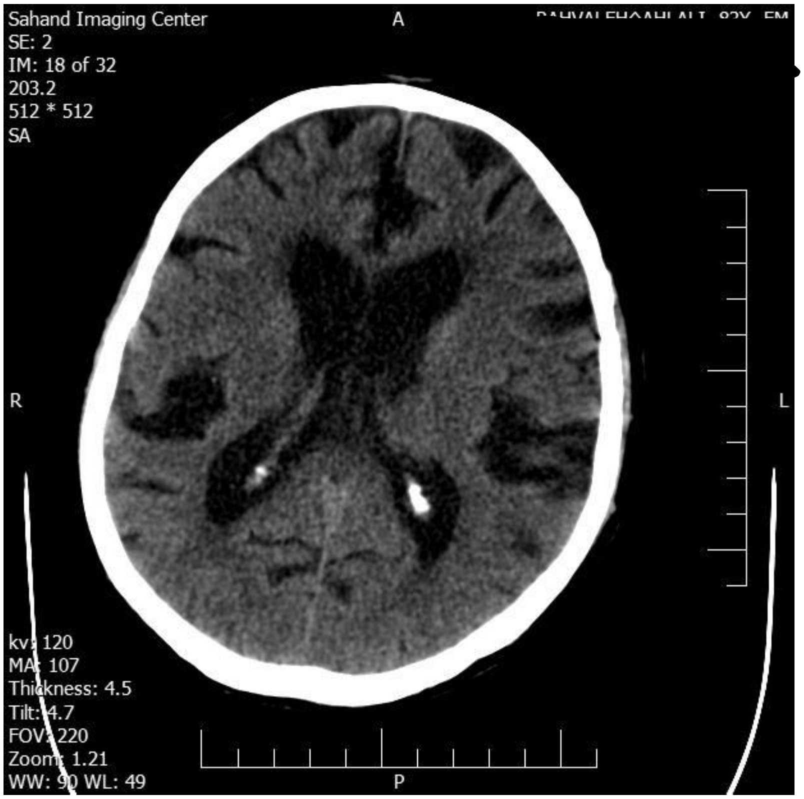
Sahand Imaging Center
SE: 2
IM: 18 of 32
203.2
512 * 512
SA
A
R
L
P
kv: 120
MA: 107
Thickness: 4.5
Tilt: 4.7
FOV: 220
Zoom: 1.21
WW: 90 WL: 49

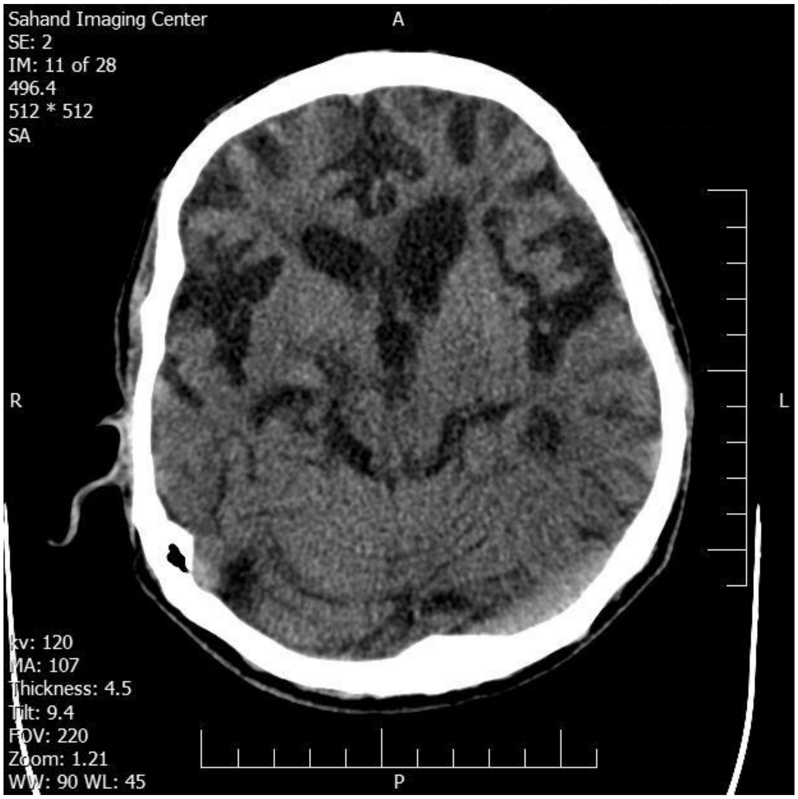
Sahand Imaging Center
SE: 2
IM: 11 of 28
496.4
512 * 512
SA
A
R
L
P
kv: 120
MA: 107
Thickness: 4.5
Tilt: 9.4
FOV: 220
Zoom: 1.21
WW: 90 WL: 45

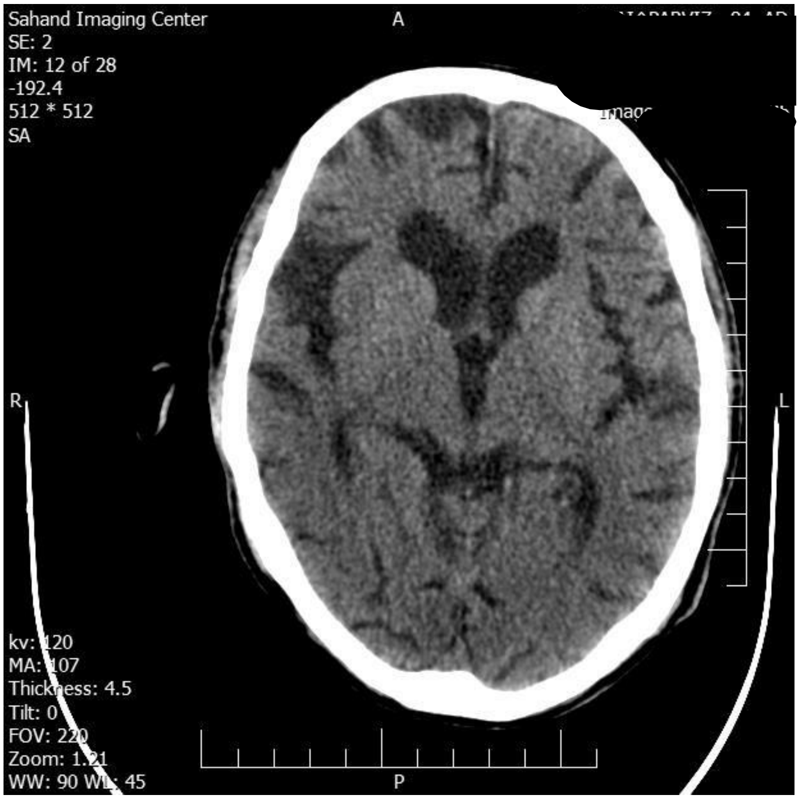
Sahand Imaging Center
SE: 2
IM: 12 of 28
-192.4
512 * 512
SA
A
[redacted]
R
L
kv: 120
MA: 107
Thickness: 4.5
Tilt: 0
FOV: 220
Zoom: 1.21
WW: 90 WL: 45
P

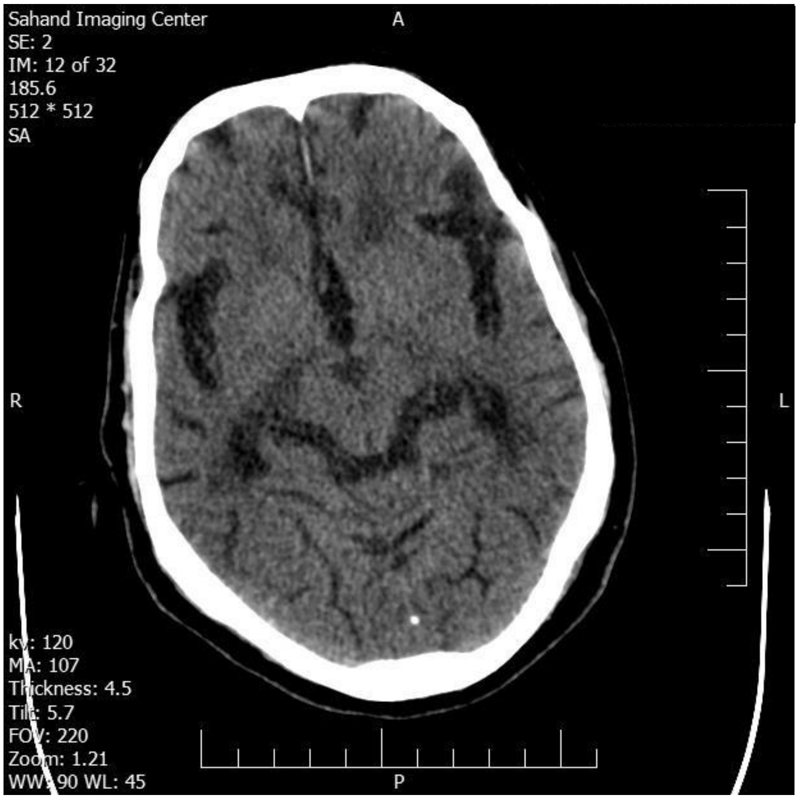
Sahand Imaging Center
SE: 2
IM: 12 of 32
185.6
512 * 512
SA
A
R
L
kv: 120
MA: 107
Thickness: 4.5
Tilt: 5.7
FOV: 220
Zoom: 1.21
WW: 90 WL: 45
P

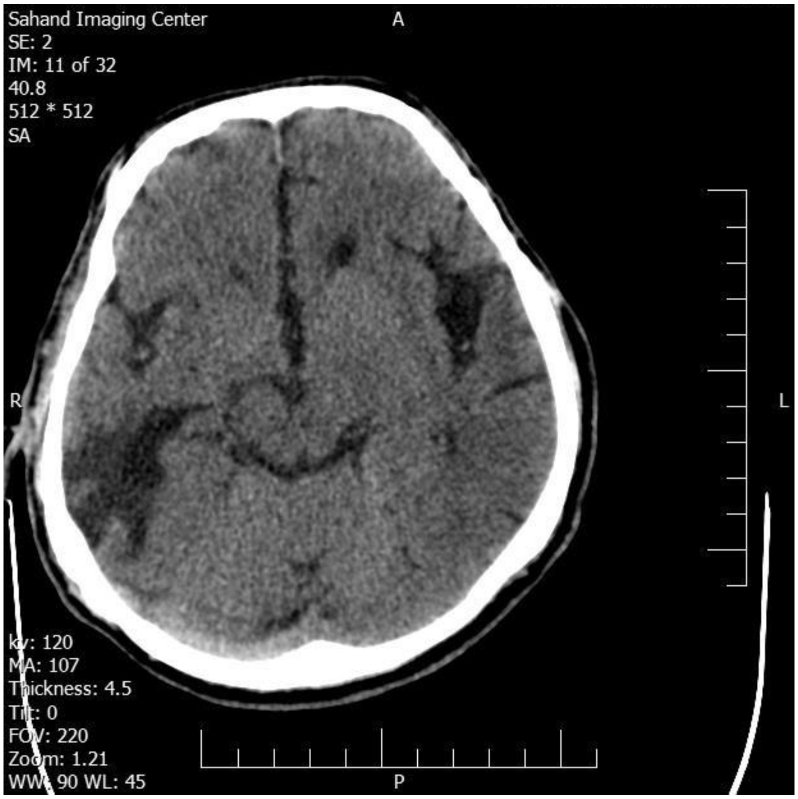
Sahand Imaging Center
SE: 2
IM: 11 of 32
40.8
512 * 512
SA
A
R
L
kv: 120
MA: 107
Thickness: 4.5
Tilt: 0
FOV: 220
Zoom: 1.21
WW: 90 WL: 45
P

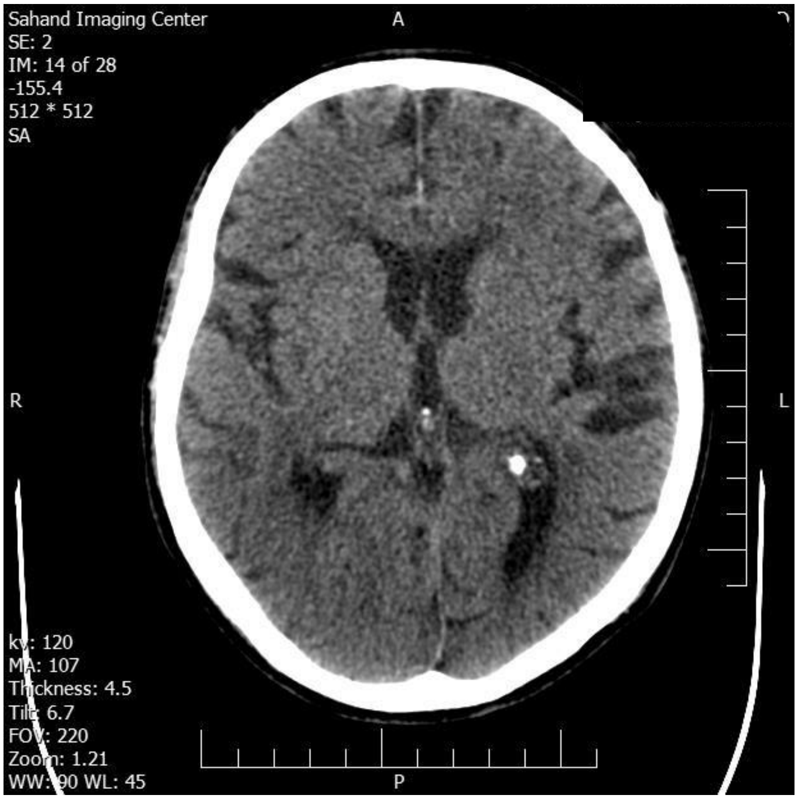
Sahand Imaging Center
SE: 2
IM: 14 of 28
-155.4
512 * 512
SA
A
R
L
kv: 120
MA: 107
Thickness: 4.5
Tilt: 6.7
FOV: 220
Zoom: 1.21
WW: 90 WL: 45
P

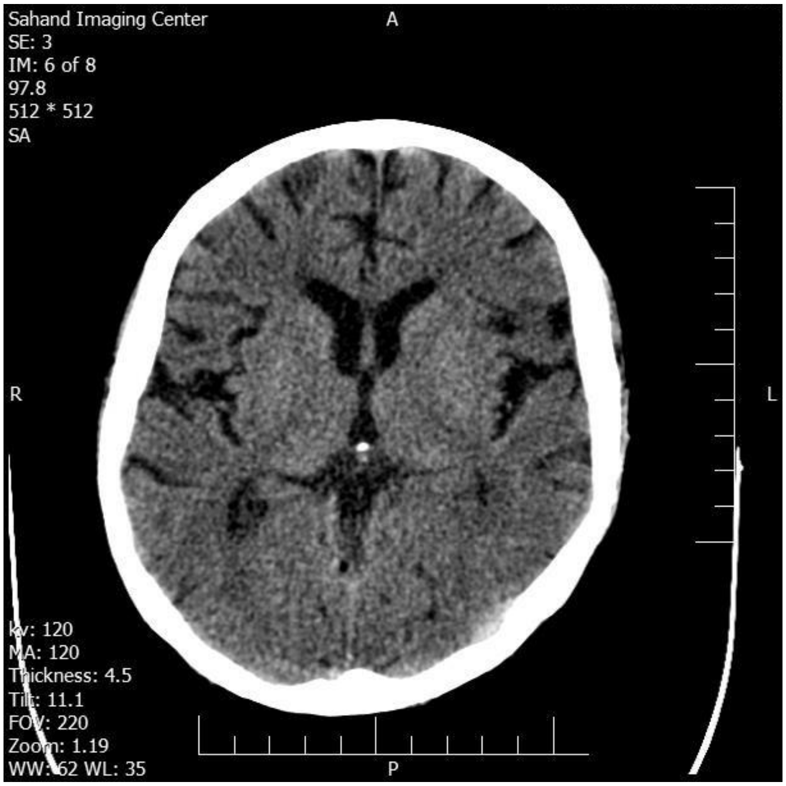
Sahand Imaging Center
SE: 3
IM: 6 of 8
97.8
512 * 512
SA
A
R
L
P
kv: 120
MA: 120
Thickness: 4.5
Tilt: 11.1
FOV: 220
Zoom: 1.19
WW: 62 WL: 35

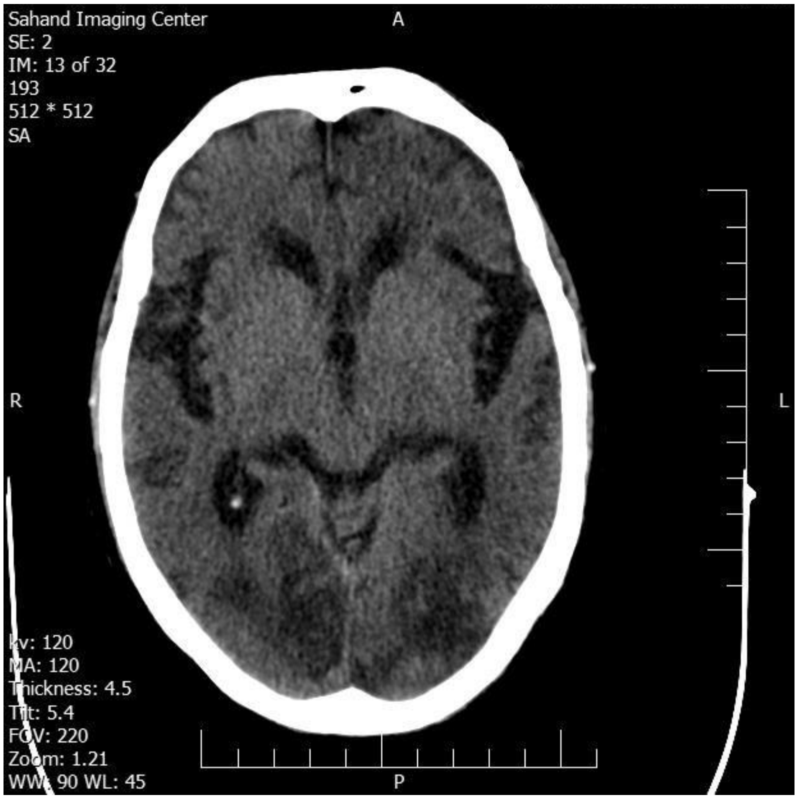
Sahand Imaging Center
SE: 2
IM: 13 of 32
193
512 * 512
SA
A
R
L
P
kv: 120
MA: 120
Thickness: 4.5
Tilt: 5.4
FOV: 220
Zoom: 1.21
WW: 90 WL: 45

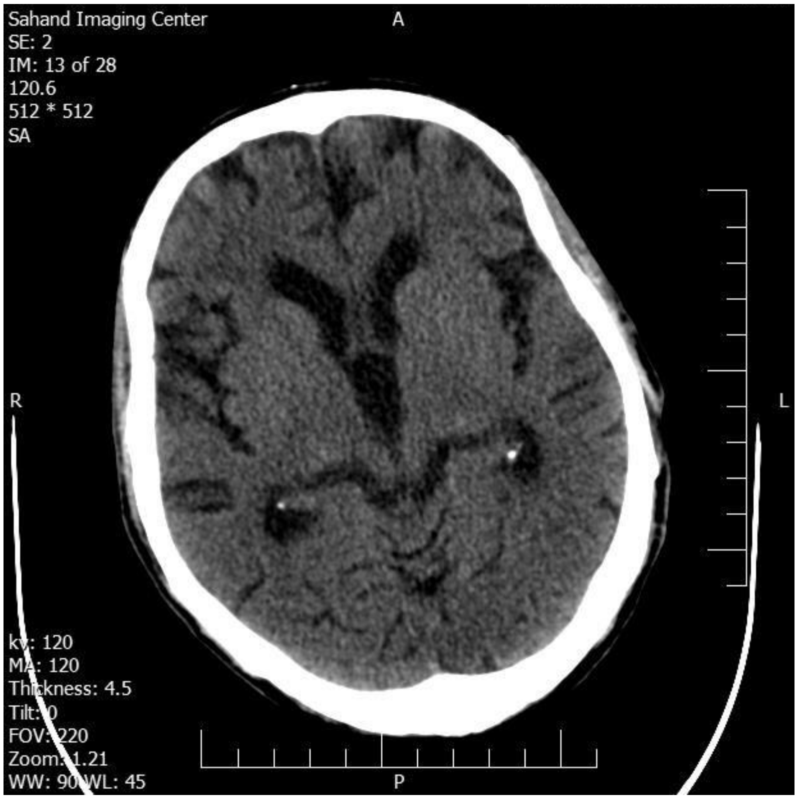
Sahand Imaging Center
SE: 2
IM: 13 of 28
120.6
512 * 512
SA
A
R
L
P
kv: 120
MA: 120
Thickness: 4.5
Tilt: 0
FOV: 220
Zoom: 1.21
WW: 90 WL: 45

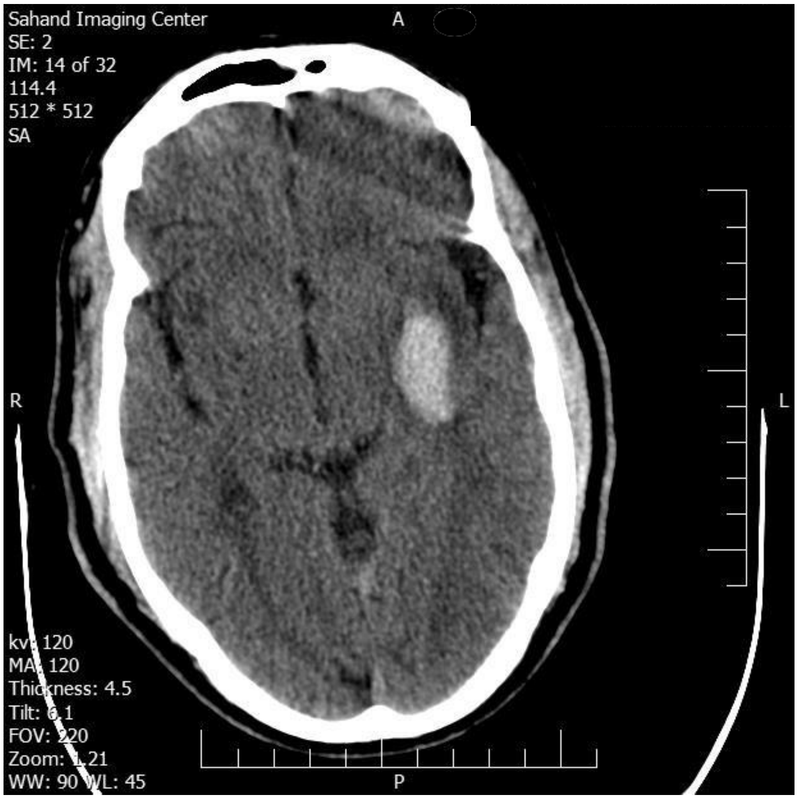
Sahand Imaging Center
SE: 2
IM: 14 of 32
114.4
512 * 512
SA
A
R
L
P
kv: 120
MA: 120
Thickness: 4.5
Tilt: 0.1
FOV: 220
Zoom: 1.21
WW: 90 WL: 45

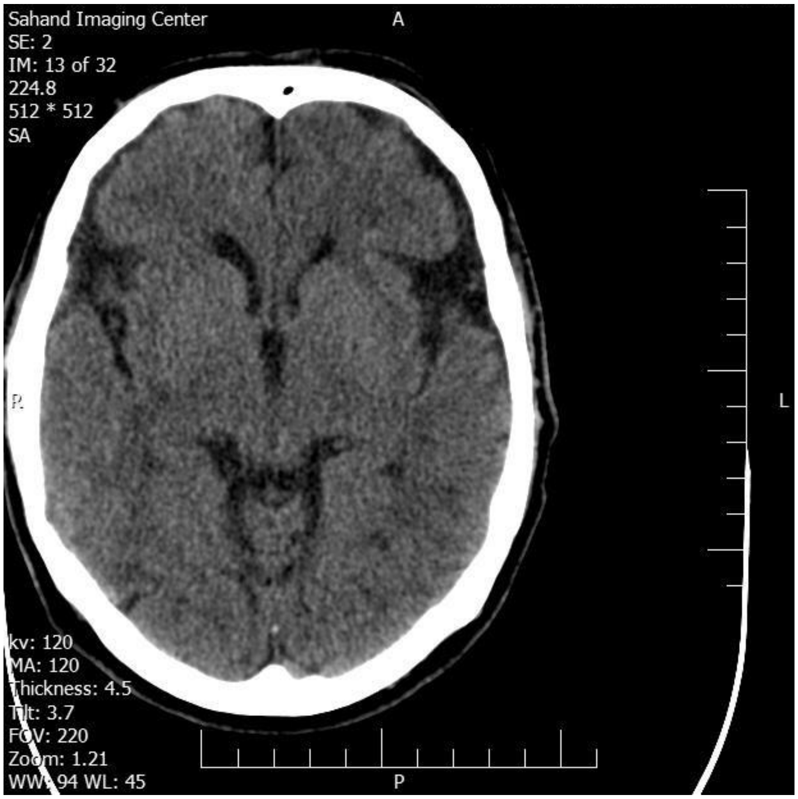
Sahand Imaging Center
SE: 2
IM: 13 of 32
224.8
512 * 512
SA
A
R
L
P
kv: 120
MA: 120
Thickness: 4.5
Tilt: 3.7
FOV: 220
Zoom: 1.21
WW: 94 WL: 45

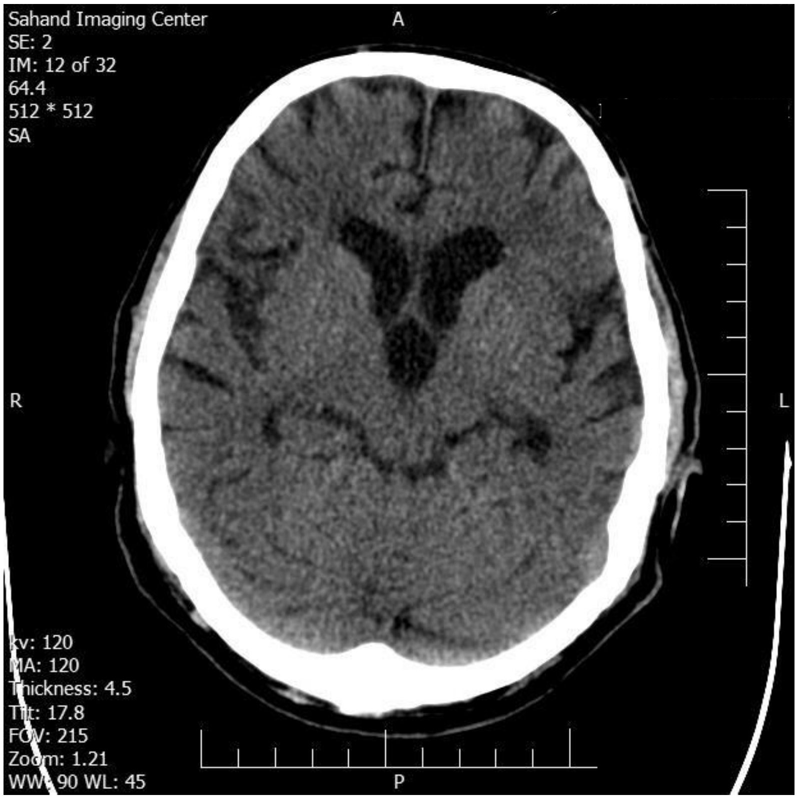
Sahand Imaging Center
SE: 2
IM: 12 of 32
64.4
512 * 512
SA
A
R
L
P
kv: 120
MA: 120
Thickness: 4.5
Tilt: 17.8
FOV: 215
Zoom: 1.21
WW: 90 WL: 45

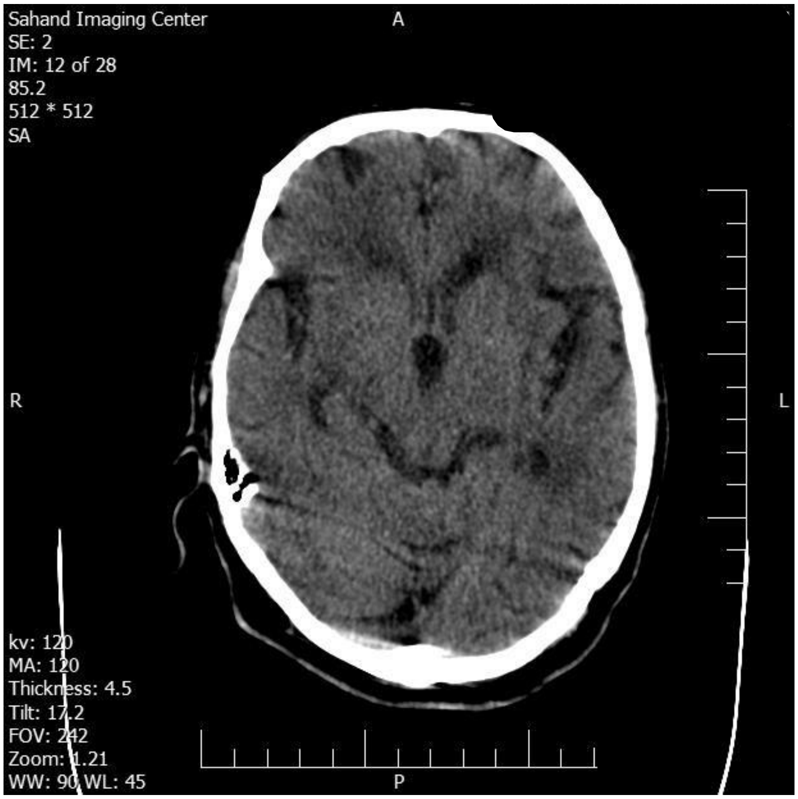
Sahand Imaging Center
SE: 2
IM: 12 of 28
85.2
512 * 512
SA
A
R
L
P
kv: 120
MA: 120
Thickness: 4.5
Tilt: 17.2
FOV: 242
Zoom: 1.21
WW: 90 WL: 45

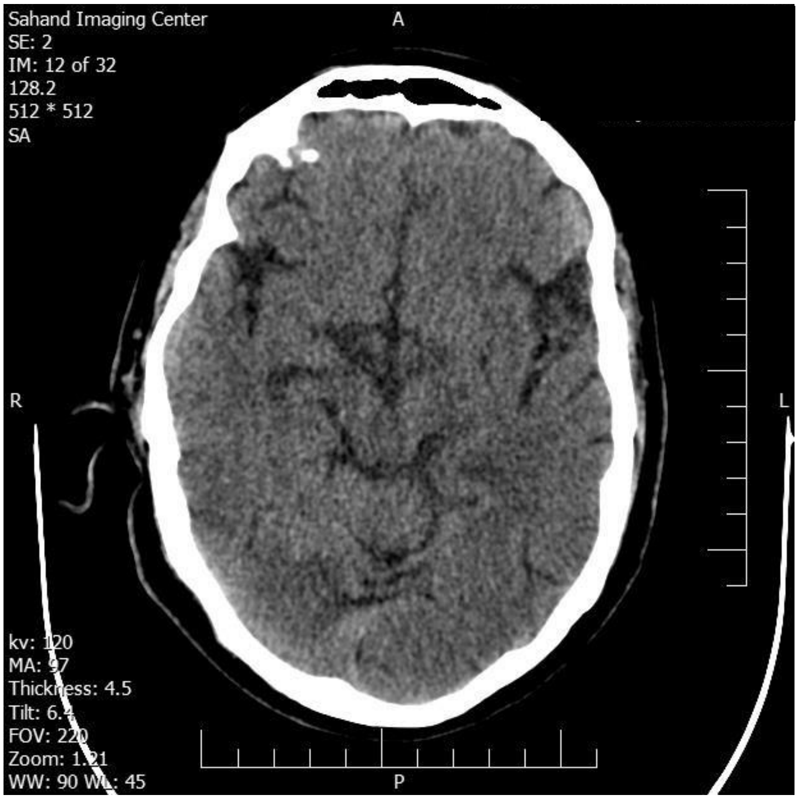
Sahand Imaging Center
SE: 2
IM: 12 of 32
128.2
512 * 512
SA
A
R
L
P
kv: 120
MA: 97
Thickness: 4.5
Tilt: 6.4
FOV: 220
Zoom: 1.21
WW: 90 WL: 45

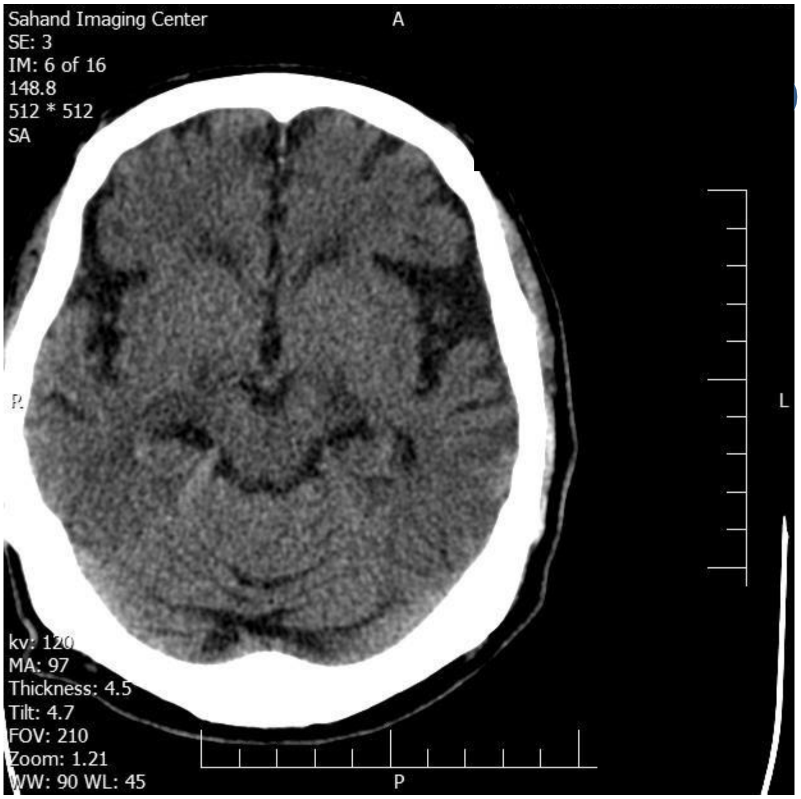
Sahand Imaging Center
SE: 3
IM: 6 of 16
148.8
512 * 512
SA
A
R
L
kv: 120
MA: 97
Thickness: 4.5
Tilt: 4.7
FOV: 210
Zoom: 1.21
WW: 90 WL: 45
P

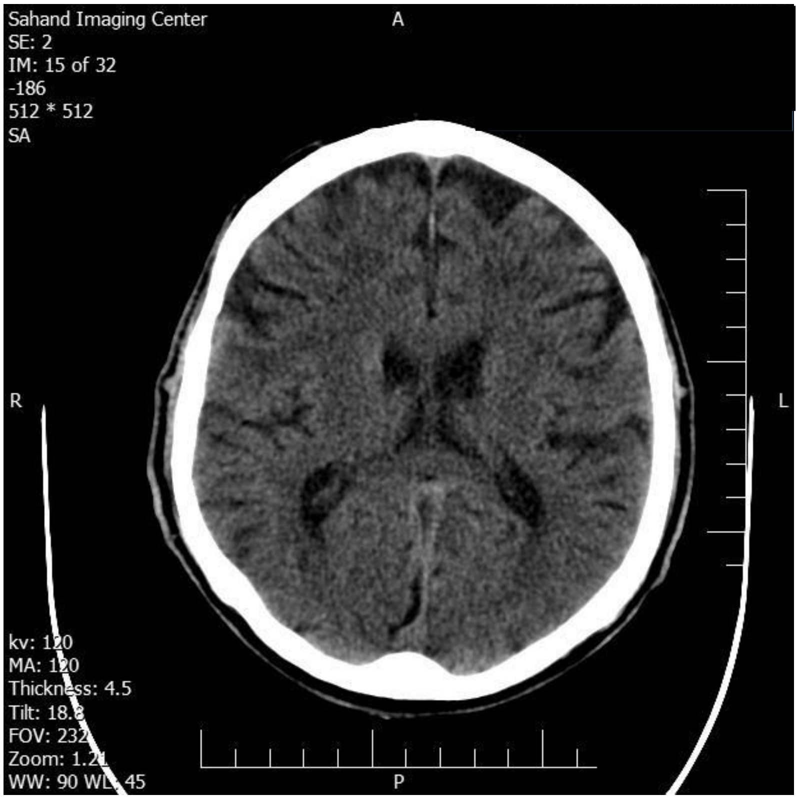
Sahand Imaging Center
SE: 2
IM: 15 of 32
-186
512 * 512
SA
A
R
L
kv: 120
MA: 120
Thickness: 4.5
Tilt: 18.8
FOV: 232
Zoom: 1.21
WW: 90 WL: 45
P

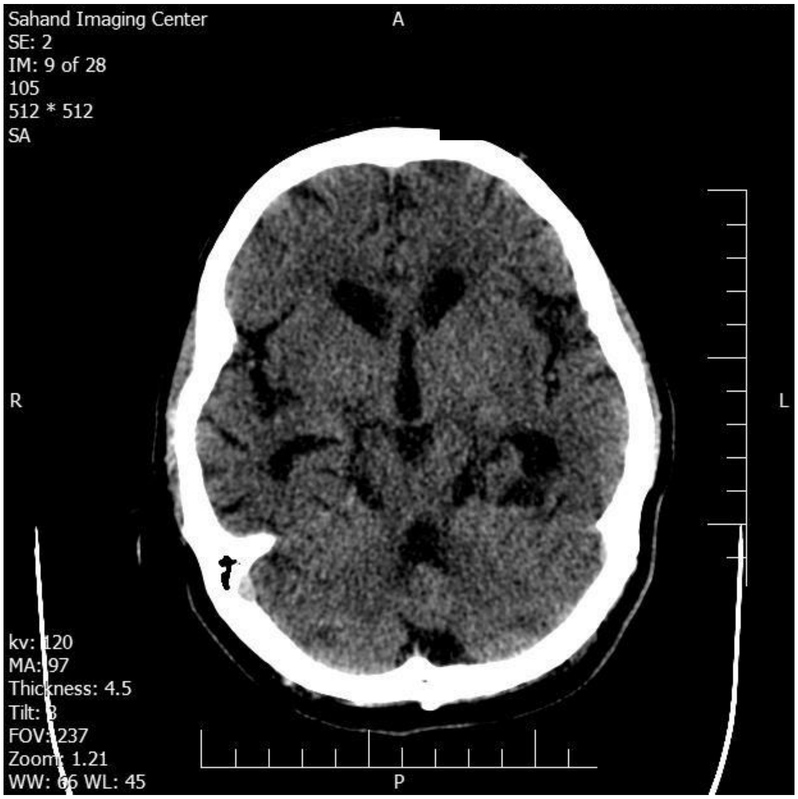
Sahand Imaging Center
SE: 2
IM: 9 of 28
105
512 * 512
SA
A
R
L
kv: 120
MA: 97
Thickness: 4.5
Tilt: 3
FOV: 237
Zoom: 1.21
WW: 66 WL: 45
P

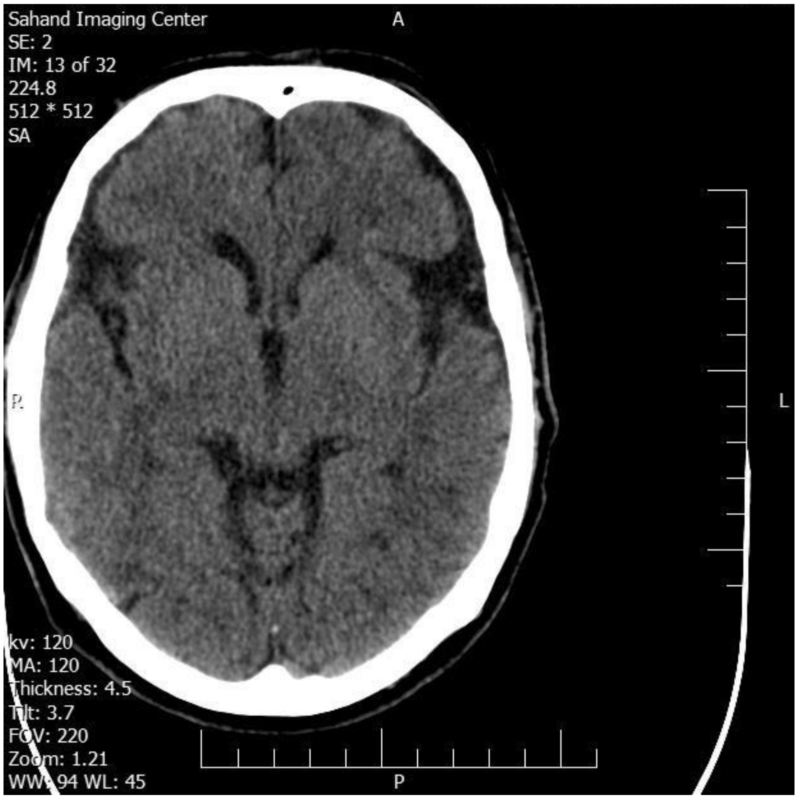
Sahand Imaging Center
SE: 2
IM: 13 of 32
224.8
512 * 512
SA
A
R
L
kv: 120
MA: 120
Thickness: 4.5
Tilt: 3.7
FOV: 220
Zoom: 1.21
WW: 94 WL: 45
P

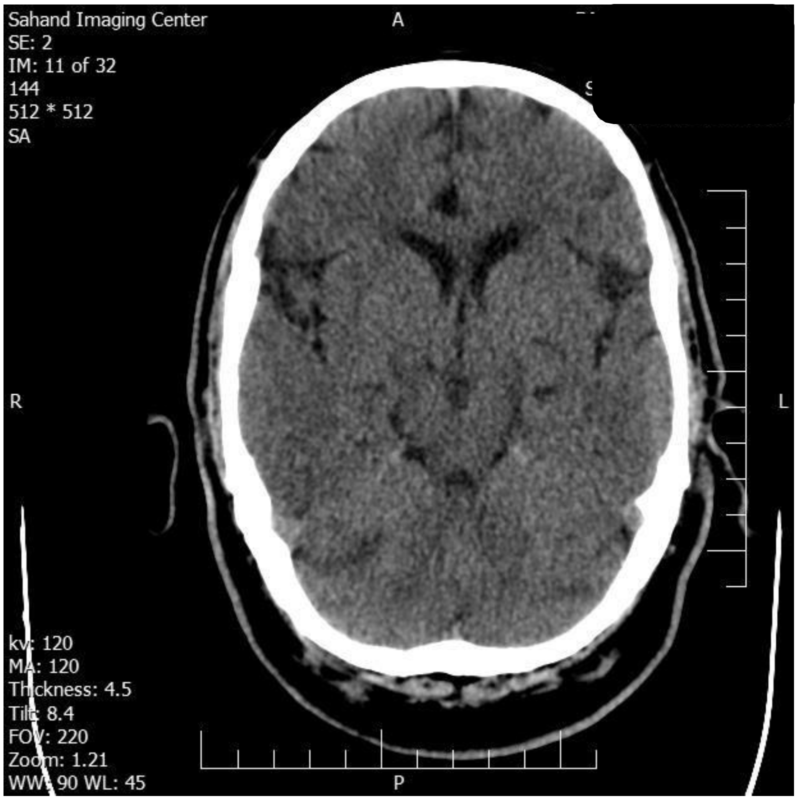
Sahand Imaging Center
SE: 2
IM: 11 of 32
144
512 * 512
SA
A
[redacted]
R
L
kv: 120
MA: 120
Thickness: 4.5
Tilt: 8.4
FOV: 220
Zoom: 1.21
WW: 90 WL: 45
P

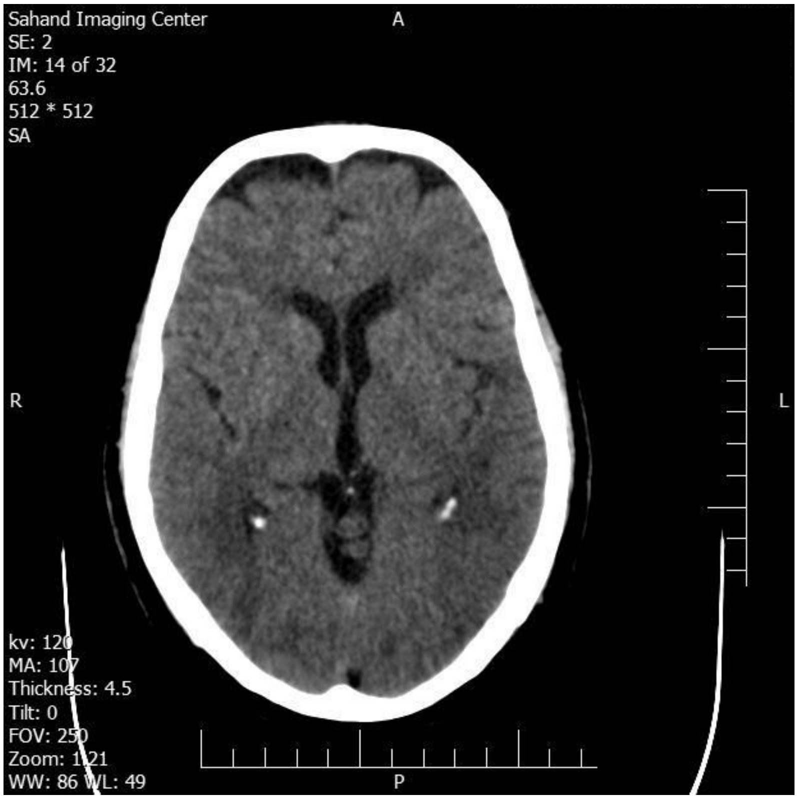
Sahand Imaging Center
SE: 2
IM: 14 of 32
63.6
512 * 512
SA
A
R
L
kv: 120
MA: 107
Thickness: 4.5
Tilt: 0
FOV: 250
Zoom: 1.21
WW: 86 WL: 49
P

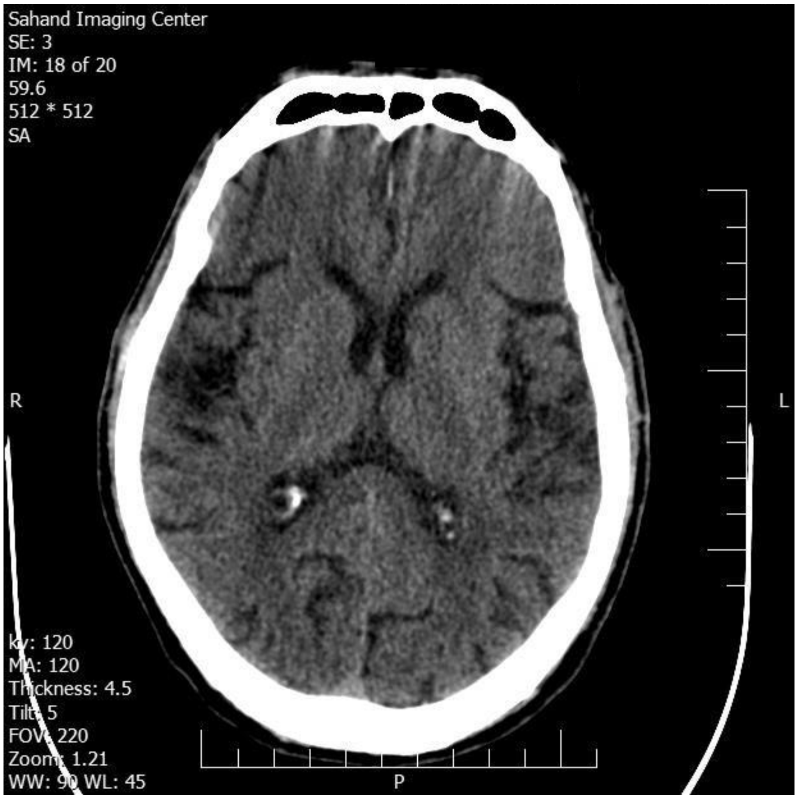
Sahand Imaging Center
SE: 3
IM: 18 of 20
59.6
512 * 512
SA
R
L
kv: 120
MA: 120
Thickness: 4.5
Tilt: 5
FOV: 220
Zoom: 1.21
WW: 90 WL: 45
P

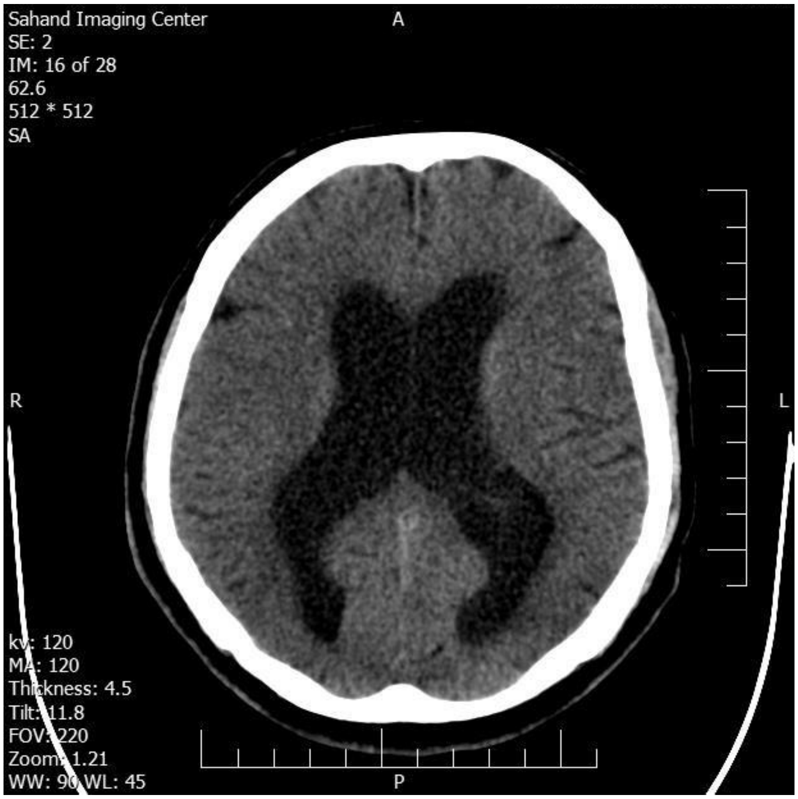
Sahand Imaging Center
A
SE: 2
IM: 16 of 28
62.6
512 * 512
SA
R
L
kv: 120
MA: 120
Thickness: 4.5
Tilt: 11.8
FOV: 220
Zoom: 1.21
WW: 90 WL: 45
P

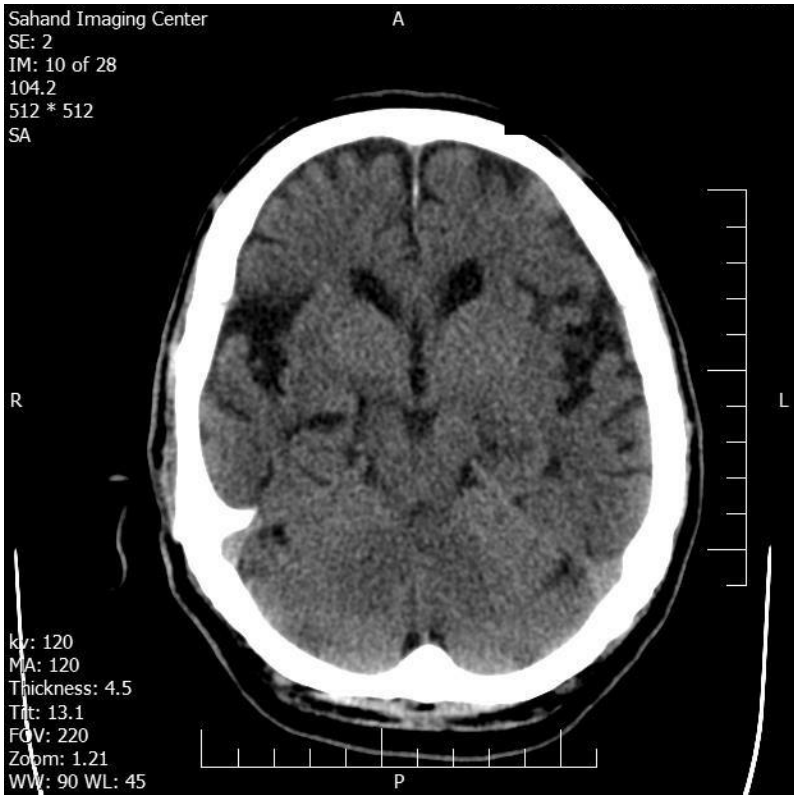
Sahand Imaging Center
SE: 2
IM: 10 of 28
104.2
512 * 512
SA
A
R
L
kv: 120
MA: 120
Thickness: 4.5
Tilt: 13.1
FOV: 220
Zoom: 1.21
WW: 90 WL: 45
P

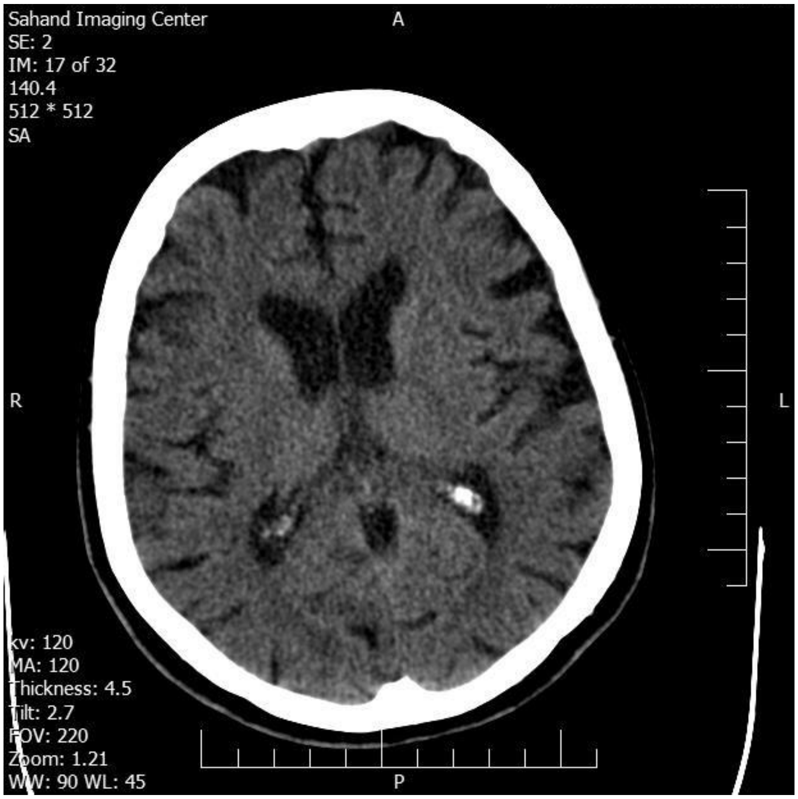
Sahand Imaging Center
SE: 2
IM: 17 of 32
140.4
512 * 512
SA
A
R
L
kv: 120
MA: 120
Thickness: 4.5
Tilt: 2.7
FOV: 220
Zoom: 1.21
WW: 90 WL: 45
P

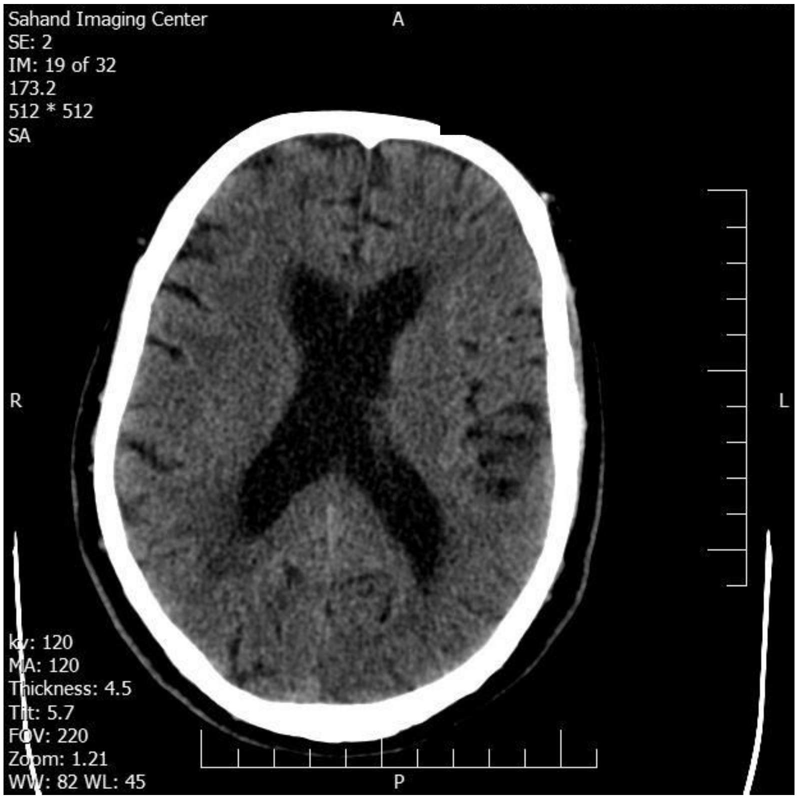
Sahand Imaging Center
SE: 2
IM: 19 of 32
173.2
512 * 512
SA
A
R
L
kv: 120
MA: 120
Thickness: 4.5
Tilt: 5.7
FOV: 220
Zoom: 1.21
WW: 82 WL: 45
P
