# Supplementary material for: Evolving evidence on a link between the ZMYM3 exceptionally long GA-STR and human cognition
Source: Sci Rep. 2020 Nov 10;10:19454. doi: 10.1038/s41598-020-76461-z (PMC7655811; doi:10.1038/s41598-020-76461-z)
Supplement: Supplementary file 2 — Supplementary Caption. [file 41598_2020_76461_MOESM2_ESM.docx]

Suppl. 1. CT-scans of a number of NCD patients.
